# First detection and identification of *Candidatus* Neoehrlichia mikurensis in South Korea

**DOI:** 10.1371/journal.pone.0209685

**Published:** 2018-12-28

**Authors:** Piyush Jha, Choon-Mee Kim, Dong-Min Kim, Na-Ra Yoon, Babita Jha, Jung Wook Park, Jae Keun Chung

**Affiliations:** 1 Deaprtment of Internal Medicine, School of Medicine, Chosun University, Gwangju, Republic of Korea; 2 Department of Premedical Science, School of Medicine, Chosun University, Gwangju, Republic of Korea; 3 Division of Infectious Disease Investigation, Health and Environment Research Institute of Gwangju City, Gwangju, Republic of Korea; Johns Hopkins University, UNITED STATES

## Abstract

*Candidatus* Neoehrlichia mikurensis (*Ca*. N. mikurensis; family *Anaplasmataceae*) is an emerging tick-borne pathogen that causes a systemic inflammatory syndrome with thrombotic complications. We report here the first identification of *Ca*. N. mikurensis in organ samples from small mammals captured in southwest South Korea. Nested PCR of *groEL* and 16S rRNA genes was used to confirm the identity of the bacteria present, and successfully amplified fragments were sequenced. All captured animals were identified as striped field mice (*Apodemus agrarius*), approximately 28.6% (4/14) and 21.4% (3/14) of which were found to be PCR-positive for *Ca*. N. mikurensis and *Anaplasma phagocytophilum*, respectively. The detection of *Ca*. N. mikurensis in these animals represents the first evidence of this pathogen in South Korea. Carriage of this bacterium by rodents highlights the need for more detailed investigation of their role in its transmission to humans.

## Introduction

*Candidatus* Neoehrlichia mikurensis (*Ca*. N. mikurensis) is an emerging tick-borne pathogen that causes a systemic inflammatory syndrome principally affecting individuals with preexisting hematologic or autoimmune diseases. As it is neither well-known nor well-recognized, *Ca*. N. mikurensis infection may be misdiagnosed as a recurrence of the underlying disease or an unrelated arteriosclerotic vascular event. This pathogen is transmitted by hard ticks of the genus *Ixodes* and is closely associated with rodents, in which transplacental transmission occurs [1].

*Ca*. N. mikurensis was first identified in the late 1990s as a novel α-proteobacterial pathogen (of the family *Anaplasmataceae*) isolated from *I*. *ricinus* in the Netherlands and Italy and a Norway rat (*Rattus norvegicus*) in China. It was initially termed *Ehrlichia*-like (or Schotti variant, *E*. *walkeri*, *Rattus-*strain) due to a divergent 16S rRNA gene sequence [2–4], but following further reports of its presence in rats and *I*. *ovatus* ticks in Japan and its passaging in laboratory rats, was described as a new species in 2004 [5]. *Ca*. N. mikurensis has been shown to be a human pathogen, and has been isolated from the blood of febrile patients across Europe and Asia. In most cases, these patients were immunocompromised due to splenectomy or immunosuppressive therapy and exhibited severe symptoms, including thrombotic events, recurrent fever lasting up to 8 months, and even death [6–8].

Several studies have identified *Ca*. N. mikurensis in questing and host-attached *I*. *ricinus* ticks in Europe [4, 9, 10]. However, potential reservoirs and vectors of this bacterium in South Korea have not yet been assessed, despite its documented presence in humans, rodents, and vectors in neighboring countries (China and Japan). Our aim was to evaluate the occurrence of this novel bacterium in the city of Gwangju, South Korea. The current work provides new data concerning the presence of this human pathogen in rodents, and constitutes the first report of *Ca*. N. mikurensis in South Korea.

## Materials and methods

### Study site and collection of rodents

Wild rodents were captured using live traps in a sylvatic habitat within an area of farmland in the west of the city of Gwangju, southwest South Korea (34°10′N, 126°55′E), during Autumn 2016 (from October through November). The sampling area consisted of a mixed stand with well-developed leaf litter layers. The live traps were placed along 5 transects (each 45 × 7 cm) spaced 150–200 m apart, depending on location, and were checked the following morning. Any small mammals caught were euthanized by inhalation of 5% isoflurane and organ samples were stored at −20°C until needed in experiments. All of the 14 wild rodents captured were identified as striped field mice (*Apodemus agrarius*). Twelve were captured in October and two in November. Each mouse was numbered for convenience during experiments and data interpretation.

### Ethics statement

This study was been approved by Chosun University Institute of Animal Care and Use Committee (CIACUC2016- S0003). It adheres to Korean Animal Protection Act (2007) Institutional Animal Care and Use (IACUC) committee guidelines and use protocol. The study was carried out on private farmland in the west of the city of Gwangju, southwest South Korea and we obtained informed verbal consent from the owner. The study only involved rodents (wild type mice; Apodemus agrarius) which is not an endangered or protected species in South Korea. Live traps were used to collect the rodents. The sampling area consisted of a mixed stand with well-developed leaf litter layers. The live traps were placed along 5 transects (each 45 × 7 cm) spaced 150–200 m apart, depending on location, and were checked the following morning. The mice were euthanized by inhalant anesthetics, carbon dioxide (CO2). Any small mammals caught were euthanized, and organ samples were stored at −20°C until needed in experiments. All the sampling procedures and experimental manipulations were closely monitored by IACUC committee members.

### DNA extraction from mouse spleen and kidney samples

Spleen and kidney samples (10 mg) from each of the 14 mice were taken from storage at −20°C, homogenized by grinding, and filtered with a sterile nylon cell strainer (70 μm; Falcon, Corning, NY, USA), before being completely lysed by proteinase K treatment and overnight incubation in a water bath. Genomic DNA was then extracted using a QIAamp DNA Blood & Tissue Mini Kit (QIAGEN, Hilden, Germany) following the manufacturer’s instructions.

### PCR amplification

Tissue samples were tested for *Ca*. N. mikurensis using nested PCR targeting a region of the *groEL* gene, which encodes a 60-kDa heat shock protein. These results were confirmed by amplification of the 16S rRNA gene. The *groEL* nested PCR was carried out with the primers GROEL 607F and GROEL 1294R for the initial amplification, and GROEL 667F and GROEL 1121R to generate a final product of 445 bp [11]. The *Ca*. Neoehrlichia-specific 16S rRNA nested PCR employed the external and internal primer pairs 16S-EC9-F/16S-EC12A-R and 16S-IS58-62f/16S-IS58-594r, respectively, yielding a final product of 488 bp [5]. A separate nested PCR specific to the *Anaplasma phagocytophilum* 16S rRNA gene was performed using the external primers AE1-F/AE1-R and internal primers AP-F/AP-R to give a final product of 926 bp [12]. *Ehrlichia chaffeensis* and *A*. *phagocytophilum* genomic DNA samples served as positive controls. Both nested PCR rounds were performed in an AB thermal cycler (Applied Biosystems, Foster City, CA, USA) with a 20-μL mixture consisting of 1 μL 10 pmol/μL primers, 10 μL master mix, 2 μL GC enhancer, 4 μL sterile distilled water, and 2 μL genomic DNA (for the first PCR) or 2 μL of the first PCR product (for the second PCR). The primer sequences and annealing temperatures used are shown in the Table 1. Amplicons were separated by electrophoresis on a 1.5% agarose gel and visualized by ethidium bromide staining.

**Table 1:** Nucleotide sequences of polymerase chain reaction primers and conditions for amplification of *Anaplasma* and *Candidatus* Neoehrlichia species genes.

Species and target genes	PCR primer sequence (5’-3’)	Annealing (°C/min)	PCR product size (bp)	References
*Anaplasma and Ehrlichia spp*. *groEL* (external primer)	GROEL -607F (5′-GAAGATGCWGTWGGWTGTACKGC-3′) GROEL 1294R (5′-AGMGCTTCWCCTTCWACRTCYTC-3′)	57	688	30
*Anaplasma and Ehrlichia spp*. *groEL* (internal primer)	GROEL- 667F (5′-ATTACTCAGAGTGCTTCTCARTG-3′) GROEL -1121R (5′-TGCATACCRTCAGTYTTTTCAAC-3′)	57	445	30
*Ca*. Neoehrlichia 16S rRNA (external primer)	16S-EC9-F (5′-TACCTTGTTACGACTT-3′) 16S-EC12A-R (5′-TGATCCTGGCTCAGAACGAACG-3′)	41	1,462	5
*Ca*. Neoehrlichia 16S rRNA (internal primer)	16S-IS58-62f (5′-GGAATAGCTGTTAGAAATGACA-3′) 16S-IS58-594r (5′-CTATCCTCTCTCGATCTCTAGTTT-3′)	54	488	5
*A*. *phagocytophilum* 16S rRNA (external primer)	AE1-F (5′-AAGCTTAACACATGCAAGTCGAA-3′) AE1-R (5′-AGTCACTGA CCCAACCTTAAATG-3′)	56	1,406	30
*A*. *phagocytophilum* *16S rRNA* (internal primer)	AP-F (5′-GTCGAACGGATTATTCTTTATAGCTTGC-3′) AP-R (5′-CCCTTCCGTTAAGAAGGATCTAATCTCC-3′)	56	926	30

### Nucleotide sequencing

**Fig 1:**
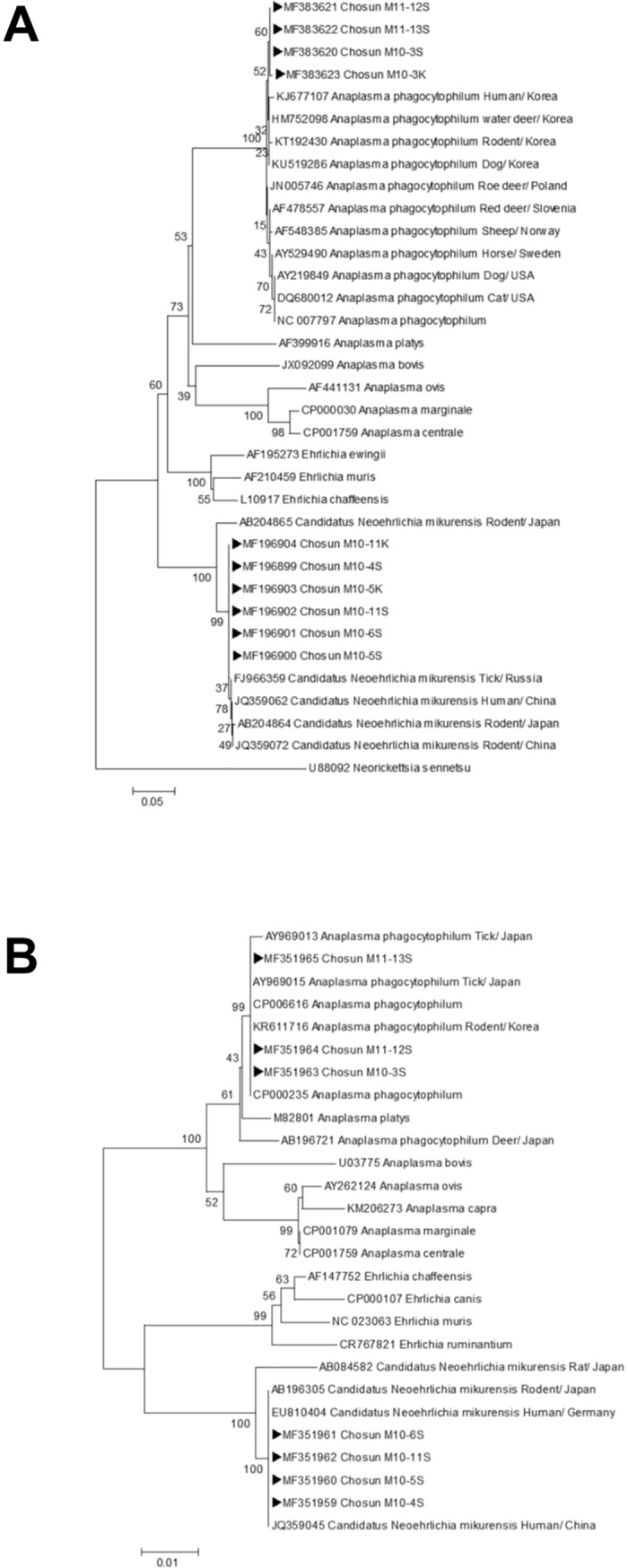
Neighbor-joining trees based on groEL (A, 445 bp) and 16S rRNA (B, 463 bp) gene sequences from GenBank and rodents having tested PCR-positive for Candidatus Neoehrlichia mikurensis and Anaplasma phagocytophilum in this study. The trees were generated in Molecular Evolutionary Genetics Analysis version 4.0 using the maximum composite likelihood method and 1,000 bootstrap replicates. Numbers next to nodes indicate the percentages of replicate trees in which the corresponding clade was recovered. Scale bars indicate 0.05 (A) and 0.01 (B) base substitutions per site. GenBank accession numbers and sources of *Ca*. N. mikurensis and *A*. *phagocytophilum* sequences are shown.

The *groEL* and 16S rRNA gene fragments amplified from positive spleen and kidney samples were purified and directly sequenced. The PCR products were visualized by electrophoresis on an ethidium bromide-stained 1.5% agarose gel. A Biosystems Veriti 96-Well Thermal Cycler (Applied Biosystems, Foster City, CA) was used for this experiment. Amplified and purified DNA was prepared for direct sequencing using a QIAquick PCR Purification Kit (Qiagen, Westburg, Netherlands) and was sequenced by dideoxy termination with an automatic sequencer (ABI Prism 3730XL DNA analyzer). Sequence homology analysis was performed by the National Center for Biotechnology Information (National Institutes of Health) BLAST network service. The resulting sequences were used in BLASTN searches of the National Center for Biotechnology Information GenBank database to identify the bacteria present. The nucleotide sequences generated in this study have been deposited in GenBank (Fig 1A and Fig 1B).

### Phylogenetic analysis

*GroEL* and 16S rRNA gene sequences were obtained from GenBank, aligned with ClustalX, and analyzed using Molecular Evolutionary Genetics Analysis version 6.0. Phylogenetic trees were constructed with the neighbor-joining method, and the percentage of replicate trees in which nodes were recovered under the bootstrap test (1,000 replicates) was calculated.

## Results

*Ca*. N. mikurensis was detected in one or more organs of 4 of the 14 mice captured. Three of the 4 *Ca*. N. mikurensis -positive animals were caught in October, and 1 was caught in November. The *groEL* nested PCR revealed 7 spleen and 3 kidney samples to be positive for *Anaplasma*/*Ehrlichia*. Sequencing identified *A*. *phagocytophilum* in 3 of these 7 spleen samples (Chosun M10-3S, M11-12S, and M11-13S) and *Ca*. N. mikurensis in the other four (Chosun M10-4S, M10-5S, M10-6S, and M10-11S).

*A*. *phagocytophilum* and *Ca*. Neoehrlichia 16S rRNA genes were detected by species-specific nested PCR. Four spleen samples were PCR-positive for *Ca*. Neoehrlichia (Chosun M10-4S, M10-5S, M10-6S, and M10-11S), and 3 for *A*. *phagocytophilum* (Chosun M10-3S, M10-12S, M11-13S). Sequencing of the PCR products confirmed the presence of *Ca*. N. mikurensis and *A*. *phagocytophilum*, respectively.

Phylogenetic trees were inferred from comparisons of *groEL (445 bp)* and 16S rRNA (463 bp) gene sequences (Fig 1A and Fig 1B). The *Ca*. N. mikurensis (488 bp) and *A*. *phagocytophilum* (926 bp) 16S rRNA gene sequences were trimmed to 463 bp to create a single tree. The *Ca*. N. mikurensis sequences derived from the spleen and kidney samples in the present study were phylogenetically close to *Ca*. N. mikurensis sequences previously isolated from rodents in China, Japan, and Russia, being grouped in the same clade. Similarly, in the phylogenies generated, the *A*. *phagocytophilum* sequences obtained here neighbored those of *A*. *phagocytophilum* previously detected in rodents from Korea.

The trees generated with *groEL* and 16S rRNA gene sequences had similar topologies and both indicated a close relationship between the bacteria examined in this investigation and other organisms identified as *Ca*. N. mikurensis. As in previous studies, *Ca*. N. mikurensis was phylogenetically distinct from other genera in the family *Anaplasmataceae* and formed a well-supported sister clade to the genus *Ehrlichia*. All of the currently available sequences from this group have been categorized together under the single candidate species *Ca*. N. mikurensis. Comparisons between the *groEL* and 16S rRNA gene sequences generated and those of specific genospecies related to *Anaplasmataceae* pathogens strongly support the *Ca*. N. mikurensis identification made here.

## Discussion

In this study, we tested small mammals to establish the occurrence of *Ca*. N. mikurensis in southwestern South Korea. As rodents have been found to harbor *Ca*. N. mikurensis, it has been suggested that they act as a reservoir of this bacterium [5, 6, 13–16]. The high infection rate observed among striped field mice in the present study corroborates this hypothesis. In a previous investigation carried out in Germany, 48 of the 91 (52.7%) small mammals tested were found to harbor *Ca*. N. mikurensis in one or more of their organs or body fluids [17]. In contrast, only 68 (8.8%) of the 771 rodents examined in a prior study based in Sweden were infected with this bacterium [15]. Similarly, a Chinese survey of 211 rodents of various species captured with snap traps revealed the rate of *Ca*. N. mikurensis carriage to be just 3.8% [18]. Nevertheless, such findings indicate that rodents play a role in the natural life cycle of *Ca*. N. mikurensis and are likely to be competent reservoir hosts of this bacterium. *Ca*. N. mikurensis has been detected in 6 rodent species in Europe (*A*. *agrarius*, *Apodemus flavicollis*, *Apodemus sylvaticus*, *Myodes glareolus*, *Microtus agrestis*, and *Microtus arvalis*) and 10 species in Asia, although infection rates vary considerably (8.3%–52.7%) between species. The bank vole *M*. *glareolus* has been identified as the most frequently infected species, with 9.1% of individuals testing positive on average. However, estimates of *Ca*. N. mikurensis prevalence in this species vary from 1.8% (in France) to 52.7% (in Germany) [1].

*Ca*. N. mikurensis was named in 2004, after its discovery in ticks and rodents on the Japanese island of Mikura-jima by using PCR targeting conserved bacterial genes, including 16S rRNA and *groEL* [5]. Transmission electron microscopy of infected rat tissue revealed small cocci in the cytoplasm of endothelial cells. Phylogenetic analyses showed this emerging zoonotic intracellular tick-borne pathogen to be a new species of the family *Anaplasmataceae*, in which it forms a distinct cluster together with the North American bacterium *Ca*. N. lotoris, which has been detected in raccoons [6, 19, 20]. Moreover, a more recent study comparing 16S rRNA and *groEL* gene sequences confirmed that organisms initially identified as “*Ehrlichia*-like” may in fact be members of the novel species *Ca*. N. mikurensis, or at least very close relations. Related species include *E*. *ruminantium*, *E*. *chaffeensis*, and *A*. *phagocytophilum* [5, 6], all of which are strict intracellular pathogens that can only be cultured in live cells. N. mikurensis retains the status “*Candidatus*” because its culture in vitro has not yet been reported.

*Ca*. N. mikurensis was first described as a human pathogen in 2010, and a total of 15 human cases of *Ca*. N. mikurensis associated disease have been reported to date in Europe and Asia, with just over half concerning apparently healthy individuals [7] and the remainder immunocompromised patients [7, 8, 21]. In a study of human *Ca*. N. mikurensis infections in China, all 7 of the patients examined exhibited relatively mild symptoms consisting of fever, headache, and malaise, and none had a history of immunocompromising illness [22]. The cells infected by Neoehrlichia bacteria in humans remain to be identified, although polymorphonuclear granulocytes and endothelial cells [6] may be involved. At present, the only diagnostic options comprise pan-bacterial PCR (targeting the 16S rRNA and *groEL* genes) followed by sequence analysis [8], and specific real-time PCR performed on whole blood, plasma, or bone marrow. Interestingly, *Ca*. N. mikurensis infection in humans appears to be associated with a high rate of vascular and thromboembolic events. Grankvist et al. reported that more than half of the affected patients (6/11) in their study developed upper- or lower-limb deep vein thrombosis [23].

The first South Korean case of human *A*. *phagocytophilum* infection occurred in 2013 [24]. Striped field mice, the dominant rodent species in South Korea and an agricultural pest, can be latently infected with various *Ehrlichia* and *Anaplasma* spp. [25, 26], and *A*. *phagocytophilum* has been identified in *Haemaphysalis longicornis*, *I*. *nipponensis*, and *I*. *persulcatus* ticks in this country [27, 28]. Moreover, previous molecular epidemiologic studies in South Korea have shown this bacterium to be present in 2.6% (5/196) of striped field mice [28, 29] and 63.6% (42/66) of Korean water deer [29]. Notably, the seroprevalence of *A*. *phagocytophilum* based on immunofluorescence tests has been estimated to be 1.8% among Korean patients with acute fever [30].

*Anaplasma* spp. and *Ca*. N. mikurensis are closely related organisms, and given the high prevalence of the latter among the wild mice examined in the present work, human infection with this pathogen in this region seems likely. Clinicians in South Korea should test for *Ca*. N. mikurensis in patients with recent tick bites seeking treatment for vascular and thromboembolic events, with a view to establishing its prevalence.

## Conclusion

In this study, 28.57% and 21.4% of the mice tested were positive for *Ca*. N. mikurensis and *A*. *phagocytophilum*, respectively. We conclude that *Ca*. N. mikurensis is widespread in the city of Gwangju, South Korea, and its relatively high prevalence in a common rodent species implies a substantial risk of infection for humans and domestic animals. Our work represents the first identification of this organism in this country, and indicates the need for more specific investigation into its importance as a human pathogen.
